# Genome sequences of 24 *Aspergillus niger sensu stricto* strains to study strain diversity, heterokaryon compatibility, and sexual reproduction

**DOI:** 10.1093/g3journal/jkac124

**Published:** 2022-05-24

**Authors:** Sjoerd J Seekles, Maarten Punt, Niki Savelkoel, Jos Houbraken, Han A B Wösten, Robin A Ohm, Arthur F J Ram

**Affiliations:** TIFN, 6708 PW, Wageningen, the Netherlands; Department Molecular Microbiology and Biotechnology, Institute of Biology, Leiden University, 2333 BE, Leiden, the Netherlands; TIFN, 6708 PW, Wageningen, the Netherlands; Microbiology, Department of Biology, Utrecht University, 3584 CH, Utrecht, the Netherlands; Department Molecular Microbiology and Biotechnology, Institute of Biology, Leiden University, 2333 BE, Leiden, the Netherlands; TIFN, 6708 PW, Wageningen, the Netherlands; Applied & Industrial Mycology, Westerdijk Fungal Biodiversity Institute, 3584 CT, Utrecht, the Netherlands; TIFN, 6708 PW, Wageningen, the Netherlands; Microbiology, Department of Biology, Utrecht University, 3584 CH, Utrecht, the Netherlands; TIFN, 6708 PW, Wageningen, the Netherlands; Microbiology, Department of Biology, Utrecht University, 3584 CH, Utrecht, the Netherlands; TIFN, 6708 PW, Wageningen, the Netherlands; Department Molecular Microbiology and Biotechnology, Institute of Biology, Leiden University, 2333 BE, Leiden, the Netherlands

**Keywords:** heterokaryon incompatibility, *Aspergillus niger*, sexual cycle, parasexual cycle, whole genome sequencing, heterozygous diploid, sclerotia, CRISPR/Cas9, mating type

## Abstract

Mating-type distribution within a phylogenetic tree, heterokaryon compatibility, and subsequent diploid formation were studied in 24 *Aspergillus niger sensu stricto* strains. The genomes of the 24 strains were sequenced and analyzed revealing an average of 6.1 ± 2.0 variants/kb between *Aspergillus niger sensu stricto* strains. The genome sequences were used together with available genome data to generate a phylogenetic tree revealing 3 distinct clades within *Aspergillus niger sensu stricto*. The phylogenetic tree revealed that both MAT1-1 and MAT1-2 mating types were present in each of the 3 clades. The phylogenetic differences were used to select for strains to analyze heterokaryon compatibility. Conidial color markers (*fwnA* and *brnA*) and auxotrophic markers (*pyrG* and *nicB*) were introduced via CRISPR/Cas9-based genome editing in a selection of strains. Twenty-three parasexual crosses using 11 different strains were performed. Only a single parasexual cross between genetically highly similar strains resulted in a successful formation of heterokaryotic mycelium and subsequent diploid formation, indicating widespread heterokaryon incompatibility as well as multiple active heterokaryon incompatibility systems between *Aspergillus niger sensu stricto* strains. The 2 vegetatively compatible strains were of 2 different mating types and a stable diploid was isolated from this heterokaryon. Sclerotium formation was induced on agar media containing Triton X-100; however, the sclerotia remained sterile and no ascospores were observed. Nevertheless, this is the first report of a diploid *Aspergillus niger sensu stricto* strain with 2 different mating types, which offers the unique possibility to screen for conditions that might lead to ascospore formation in *A. niger.*

## Introduction

Filamentous fungi, and more specifically *Aspergillus* species, are known to propagate mainly via asexual reproduction. For many *Aspergillus* species no sexual cycle is found (Samson *et al.* 2014). In fact, researchers used to believe that meiosis was rendered impossible in certain strictly asexual *Aspergillus* species such as *Aspergillus fumigatus*, *Aspergillus flavus*, *Aspergillus terreus*, and *Aspergillus niger* (Geiser *et al.* 1996). However, more recent studies and the rise of next generation sequencing revealed that *Aspergillus* species which are seemingly without a sexual cycle have the genetic information indicating that they could be able to propagate using meiosis (Dyer *et al.* 2003). Around 31% of all accepted aspergilli have been proven to reproduce sexually, with 19 species being heterothallic (Houbraken *et al.* 2020). The currently available (genomic) research data suggests that sexual reproduction within aspergilli still occurs more often than is currently shown *in vitro* (Ojeda-López *et al.* 2018). One such *Aspergillus* species without a known sexual cycle is *A. niger* (Houbraken and Dyer 2015).

The name *A. niger* has been used in a broad sense throughout literature to refer to species belonging to *Aspergillus* section *Nigri*, the “*A. niger* aggregate,” the “black aspergilli,” “the *A. niger* clade,” or the “*Aspergillus niger* group” (Abarca *et al.* 2004; Ferracin *et al.* 2009; Nielsen *et al.* 2009; da Silva *et al.* 2020). However, *A. niger* is also referred to as a species within *Aspergillus* section *Nigri.* Therefore, it became a necessity to define strains strictly belonging to the species *A. niger* specifically as *A. niger sensu stricto* strains to inform the reader about the exclusion of other black aspergilli part of *Aspergillus* section *Nigri*, such as *Aspergillus neoniger*, *Aspergillus welwitschiae*, or *Aspergillus luchuensis.* The filamentous fungus *A. niger sensu stricto* is a well-known producer of enzymes and organic acids and has been industrially and biotechnologically relevant for over 100 years (Pel *et al.* 2007; Andersen *et al.* 2011; Cairns *et al.* 2018). Functional sexual reproduction in industrially relevant fungi can benefit industry greatly, as it can be a useful tool in strain improvement (Kück and Böhm 2013). Genetic alterations resulting from sexual recombination are not considered genetic manipulation, making the methodology viable for strictly non-GMO strains (Garrigues *et al.* 2021). Successful studies revealing in vitro sexual reproduction in industrially important fungi, such as *Trichoderma reesei* and *Penicillium chrysogenum*, have been reported (Seidl *et al.* 2009; Böhm *et al.* 2013).

Research on heterokaryon incompatibility in ascomycetes and the involved *het* and *vic* genes has been mostly explored in *Neurospora crassa* and *Podospora anserina* (Saupe 2000). Many heterokaryon incompatibility systems have been described to date in these fungi (Gonçalves and Glass 2020). All strains that show heterokaryon compatibility with each other are considered part of the same vegetative compatibility group. Heterokaryon incompatibility in *N. crassa* and *P. anserina* generally requires an interaction between 2 proteins. Heterokaryon incompatibility occurs when different genetic versions exist within the fungus of the *het* gene or its partner, meaning that the fungus is heteroallelic for this region, resulting in incompatibility and subsequently cell death (Fedorova *et al.* 2005). In most currently described heterokaryon incompatibility systems at least one of the 2 proteins contains a HET domain (Paoletti and Clavé 2007). The HET domain is defined as a region containing 3 conserved amino acid blocks (Smith *et al.* 2000). This is true for the *het-c/pin-c* and *het-6/un-24* systems in *N. crassa* (Smith *et al.* 2000; Kaneko *et al.* 2006) as well as the *het-c/het-d* and *het-c/het-e* systems in *P. anserina* (Espagne *et al.* 2002). However, recent studies have revealed the existence of additional heterokaryon incompatibility systems that do not require proteins containing HET domains, such as *sec-9*/*plp-1* and *rcd-1* (Heller *et al.* 2018; Daskalov *et al.* 2019).

In *Aspergillus* section *Nigri*, anastomosis and subsequently plasmogamy leads to cell death in almost all cases when parasexual crosses are attempted, unless the nuclei are isogenic (van Diepeningen *et al.* 1997; Pál *et al.* 2007). A hypothetical purpose for this phenomenon of vegetative incompatibility has been proposed and states that the organism could benefit by blocking transfer of viruses when the fungus is limited to self-mating (van Diepeningen *et al.* 1998). The mechanisms behind self-recognition and subsequent heterokaryon compatibility or heterokaryon incompatibility are poorly understood in aspergilli. Previous research concluded that heterokaryon incompatibility genes in *A. niger* differs from those observed in *N. crassa* and *P. anserina* (van Diepeningen *et al.* 2009). The ortholog of the *het-C* from *N. crassa* is present in *A. niger*. When the *het-C* variant from *N. crassa* was introduced into *A. niger* an abortive phenotype was observed, indicating that the *het-C* gene of *N. crassa* functions as incompatibility gene in *A. niger.* However, the *het-C* ortholog present in *A. niger* does not seem to vary between *A. niger* strains CBS513.88 and ATCC1015 even though these strains are vegetatively incompatible. Therefore, it is unlikely that the *het-C* gene functions as a heterokaryon incompatibility gene in *A. niger.* Consequently, the widespread heterokaryon incompatibility observed in *A. niger* seems to be potentially mediated by different genes than in *N. crassa.*

**Fig. 1.**
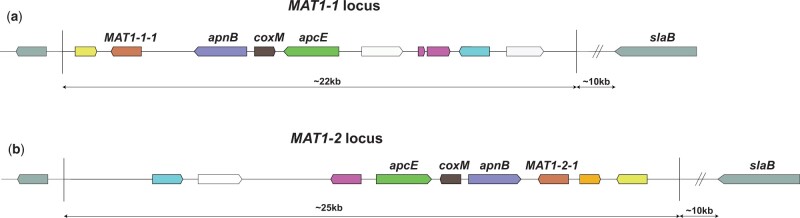
Schematical overview of the *MAT1-1* and *MAT1-2* loci in *A. niger*. Conserved genes between the 2 MAT loci have been color coded. White genes are not conserved between the 2 loci and gray genes are positioned outside of the 2 MAT loci. In red, the mating-type genes *MAT1-1-1* and *MAT1-2-1*. These genes are transcription factors, where *MAT1-1-1* contains an “alpha1 HMG-box” domain and *MAT1-2-1* contains a high mobility group (HMG) domain. In aspergilli, these mating-type genes are normally flanked by the DNA lyase *apnB* and a cytoskeleton assembly control factor *slaB*. However, in *A. niger* the *slaB* gene is located more than 10-kb downstream of the MAT genes. a) Mating-type locus *MAT1-1*. This mating-type locus appears to have a flipped orientation when compared to the *MAT1-2* locus and when compared to *MAT1-1* and *MAT1-2* loci of other aspergilli. b) Mating-type locus *MAT1-2*. Only recently described in the *A. niger* neotype strain CBS554.65, the orientation of the genes in this locus corresponds with expectations based on the *MAT1-1* and *MAT1-2* locus organization of other aspergilli. The mating-type loci, the individual genes, and their possible functions in *A. niger* have been discussed more extensively in a recent study (Ellena *et al.* 2021).

Certain filamentous fungi are homothallic and therefore able to undergo a sexual cycle with itself, such as *Aspergillus nidulans* (Paoletti *et al.* 2007). In contrast, sexual reproduction in heterothallic ascomycetes requires the crossing of strains with 2 different mating types (Coppin *et al.* 1997). Screening natural isolates of a heterothallic species for the distribution of the MAT1-1 or the MAT1-2 locus would indicate whether sexual propagation still occurs in nature. MAT loci contain up to about 19 genes, of which the presence of the MAT transcription factors (either the *MAT1-1-1* gene or the *MAT1-2-1* gene) defines the mating type (Fig. 1). Sexual reproduction of ascomycetes is mediated by the mating-type genes and results in asci wherein ascospores are formed. The formation of asci and ascospores by aspergilli occurs inside cleistothecia, and in aspergilli from sections *Flavi* and *Nigri* these cleistothecia are formed inside a stroma within sclerotia (Dyer and O’Gorman 2012). It was inside sclerotia where the first products of sexual recombination have been found after prolonged incubation times for *A. tubingensis* (Horn *et al.* 2013), *Aspergillus parasiticus* (Horn, Ramirez-Prado, *et al.* 2009), and *A.*  *flavus* (Horn, Moore, *et al.* 2009). Therefore, identifying the appropriate environmental conditions needed for sclerotia formation is considered a first-step prerequisite for finding ascospore formation.

In this work, we present a detailed analysis of 24 *A. niger sensu stricto* genomes of which 12 contain a MAT1-2 locus, first reported by Ellena *et al.* (2021). In addition, these genomes were used to construct a phylogenetic tree and subsequently perform parasexual crosses between the strains. One of these crosses resulted in a stable diploid strain containing both mating-type loci of *A. niger*. These strains and genomes sequences provide a unique opportunity to further study heterokaryon incompatibility and sexual reproduction in *A. niger*.

## Materials and methods

### Strains, media, cultivation conditions, and conidia harvesting

The strains sequenced in this study are listed in Table 1. The mutant strains made in this study are listed in Table 2. Strains are cultivated on minimal medium (MM) supplemented with 10 mM uridine and 20 µM nicotinamide when required, prepared as described previously (Arentshorst *et al.* 2012), for 7 days at 30°C unless noted otherwise. Conidia were harvested in physiological salt buffer containing Tween 80 (0.9% NaCl, 0.02% Tween 80 in demi water) and filtrated using sterile filters (Amplitude Ecocloth, CONTEC) to separate them from mycelium fragments.

**Table 1.** *Aspergillus niger sensu stricto* strains sequenced in this study.

CBS number	Synonyms	DTO number	Country of origin	Isolated from	Mating type
CBS 112.32		DTO 028-I3	Japan	Unknown	MAT1-1
CBS 113.50	NRRL 334, ATCC 6275	DTO 008-C3	Unknown	Leather	MAT1-2
CBS 118.52		DTO 058-I1	Unknown	Unknown	MAT1-2
CBS 124.48		DTO 029-B1	Unknown	Unknown	MAT1-2
CBS 131.52	NRRL 334, ATCC 6275	DTO 029-C3	Unknown	Leather	MAT1-1
CBS 630.78	NRRL 1956	DTO 067-H7	South Pacific Islands	Army equipment	MAT1-2
CBS 769.97	NRRL 334, ATCC 6275	DTO 367-D1	Unknown	Leather	MAT1-1
CBS 115988	NRRL 3112	DTO 059-C7	Unknown	Unknown	MAT1-1
CBS 115989	NRRL 3122	DTO 367-D6	Unknown		MAT1-1
CBS 133816	IBT 24631	DTO 316-E3	Denmark	Black pepper	MAT1-2
CBS 147320		DTO 096-A7	Australia	Grape	MAT1-1
CBS 147321		DTO 096-A9	Norway	Arctic soil	MAT1-2
CBS 147322		DTO 096-C6	Brazil	Coffee	MAT1-2
CBS 147323		DTO 096-D7	Turkey	Raisin	MAT1-2
CBS 147324		DTO 096-E1	Unknown	Unknown	MAT1-2
CBS 147343		DTO 291-B7	Thailand	Coffee bean	MAT1-1
CBS 147344		DTO 293-G7	Thailand	Coffee beans (Robusta)	MAT1-2
CBS 147345		DTO 316-E4	United States	Unknown	MAT1-1
CBS 147346		DTO 321-E6	The Netherlands	CF patient material	MAT1-2
CBS 147347		DTO 326-A7	The Netherlands	Petridish; soft drink factory	MAT1-1
CBS 147352		DTO 368-I1	Mexico	Air next to bottle blower	MAT1-1
CBS 147353		DTO 368-I6	Italy	Foods factory of Sanquinetto	MAT1-1
CBS 147371		DTO 096-A5	India	Green coffee bean	MAT1-1
CBS 147482		DTO 175-I5	Portugal	Surface water	MAT1-2

**Table 2.** Mutant *A. niger* strains made in this study.

Strain name	Mutations	Description	Parental strain
NS1	*pyrG^−^*, *fwnA^−^*	Fawn colored conidia, uridine deficient (needs supplement)	CBS 112.32
NS2	*pyrG^−^*, *fwnA^−^*	Fawn colored conidia, uridine deficient (needs supplement)	CBS 118.52
NS3	*nicB^−^*, *brnA^−^*	Brown colored conidia, nicotinamide deficient (needs supplement)	CBS 147371
NS4	*pyrG^−^*, *fwnA^−^*	Fawn colored conidia, uridine deficient (needs supplement)	CBS 147323
NS5	*pyrG^−^*, *fwnA^−^*	Fawn colored conidia, uridine deficient (needs supplement)	CBS 147324
NS6	*nicB^−^*, *brnA^−^*	Brown colored conidia, nicotinamide deficient (needs supplement)	CBS 147482
NS7	*pyrG^−^*, *fwnA^−^*	Fawn colored conidia, uridine deficient (needs supplement)	CBS 133816
NS8	*pyrG^−^*, *fwnA^−^*	Fawn colored conidia, uridine deficient (needs supplement)	CBS 147347
NS9	*pyrG^−^*, *fwnA^−^*	Fawn colored conidia, uridine deficient (needs supplement)	CBS 147352
NS10	*pyrG^−^*, *fwnA^−^*	Fawn colored conidia, uridine deficient (needs supplement)	CBS 147353
NS11	*nicB^−^*, *brnA^−^*	Brown colored conidia, nicotinamide deficient (needs supplement)	CBS 147343
SJS111	*nicB^−^*, *brnA^−^*	Brown colored conidia, nicotinamide deficient (needs supplement)	CBS 147323
SJS112	*nicB^−^*, *brnA^−^*	Brown colored conidia, nicotinamide deficient (needs supplement)	CBS 118.52
SJS113	*nicB^−^*, *brnA^−^*	Brown colored conidia, nicotinamide deficient (needs supplement)	CBS 112.32
SJS114	*nicB^−^*, *brnA^−^*	Brown colored conidia, nicotinamide deficient (needs supplement)	CBS 147347
SJS150.1		Heterozygous diploid strain containing 2 mating-type loci	SJS114 and NS4
SJS150.2		Heterozygous diploid strain containing 2 mating-type loci	SJS114 and NS4
SJS150.3		Heterozygous diploid strain containing 2 mating-type loci	SJS114 and NS4
SJS151.1		Heterozygous diploid strain containing 2 mating-type loci	SJS111 and NS8
SJS151.2		Heterozygous diploid strain containing 2 mating-type loci	SJS111 and NS8
SJS151.3		Heterozygous diploid strain containing 2 mating-type loci	SJS111 and NS8

### Whole genome sequencing

A total of 24 *A.*  *niger* strains were obtained from the CBS culture collection housed at the Westerdijk Fungal Biodiversity Institute, Utrecht, the Netherlands. These strains were identified as *A. niger sensu stricto* based on partial calmodulin gene sequencing (Samson *et al.* 2007). The strains were grown on malt extract agar (MEA, Oxoid) for 7 days and subsequently conidia were harvested. Liquid cultures containing complete medium (CM) (Arentshorst *et al.* 2012) were inoculated with conidia suspension and grown overnight at 30°C. Genomic DNA (gDNA) was isolated using a chloroform/phenol-based genome extraction method (Arentshorst *et al.* 2012). The gDNA was subsequently purified using the DNA purification kit NucleoSpin Plant II (Macherey-Nagel). This gDNA was sequenced at the Utrecht Sequencing Facility (USEQ) using Illumina NextSeq 500 paired-end technology. Raw sequence files were trimmed on both ends when quality was lower than 15 using bbduk from the BBMap tool suite (BBmap version 37.88; https://sourceforge.net/projects/bbmap/). The trimmed reads were assembled with SPAdes v3.11.1 applying kmer lengths of 21, 33, 55, 77, 99, and 127 (Bankevich *et al.* 2012). Sequences (scaffolds) shorter than 1,000 bp were removed from the assembly. Genes were predicted with AUGUSTUS version 3.0.3 (Stanke *et al.* 2006) using the provided parameter set for *A. nidulans* and the publicly available *A. niger* ATCC1015 transcriptome reads (SRR6012879) were used as an aid in gene prediction. Functional annotation of the predicted genes was performed as previously described (De Bekker *et al.* 2017) to assign a putative function to the genes. The assemblies and gene predictions are available from NCBI GenBank under BioProject ID PRJNA743902. Furthermore, the annotated genomes can be analyzed interactively on https://fungalgenomics.science.uu.nl/.

### Genomic differences between genomes

The genome comparisons were done using either the publicly available assembly of strain NRRL3 (Aguilar-Pontes *et al.* 2018), or the assembly of the newly sequenced strain CBS147323 as reference. The reads of the other 23 or 24 strains were aligned to these references. Genomic variants including single-nucleotide polymorphisms (SNPs) as well as indels were identified and their impact on the predicted genes was determined. The reads were aligned to the reference assemblies using Bowtie 2 version 2.4.2. (Langmead and Salzberg 2012). The resulting SAM files were provided with read groups and subsequently transformed to BAM files and sorted using SAMtools (Li *et al.* 2009). Duplicates were marked and subsequent variant calling was done using GATK HaploTypeCaller version 4.1.4.1. (Poplin *et al.* 2017), resulting in VCF files describing the genomic variants. Lastly, SnpEff and SnpSift (Cingolani, Patel, *et al.* 2012; Cingolani, Platts, *et al.* 2012) were used to determine the location of SNPs and their predicted impact in regard to the genes. Visualization and manual inspection of genomic variants were done using the Integrative Genome Viewer (Robinson *et al.* 2011).

### Construction of phylogenetic trees based on conserved proteins

The sequences of the predicted proteins of the 24 strains were used to construct phylogenetic trees. In addition, for the construction of the phylogenetic tree of Aspergillus section *Nigri*, we collected the protein files of *A.*  *welwitschiae* CBS139.54b (Vesth *et al.* 2018), *Aspergillus piperis* CBS112811 (Vesth *et al.* 2018), *A.*  *luchuensis* IFO4308 (Futagami *et al.* 2011), *Aspergillus eucalypticola* CBS122712 (Vesth *et al.* 2018), *Aspergillus costaricaensis* CBS115574 (Vesth *et al.* 2018), *Aspergillus tubingensis* WU-2223L, *A.*  *neoniger* CBS115656 (Vesth *et al.* 2018), *Aspergillus vadensis* CBS113365 (Vesth *et al.* 2018), *Aspergillus carbonarius* ITEM5010 (de Vries *et al.* 2017), *Aspergillus sclerotioniger* CBS115572 (Vesth *et al.* 2018), *Aspergillus ibericus* CBS121593 (Vesth *et al.* 2018), *Aspergillus japonicus* CBS114.51 (Vesth *et al.* 2018), *Aspergillus fijiensis* CBS313.89 (Vesth *et al.* 2018), *Aspergillus brunneoviolaceus* CBS621.78 (Vesth *et al.* 2018), *Aspergillus aculeatus* ATCC16872 (de Vries *et al.* 2017), *A.*  *fumigatus* Af293 (Nierman *et al.* 2005), *Aspergillus oryzae* RIB40 (Machida *et al.* 2005), and *Aspergillus nidulans* FGSCA4 (Galagan *et al.* 2005).

For the creation of a *A. niger sensu stricto*-specific phylogenetic tree 9 other publicly available strains were used, namely: *A. niger* ATCC1015 (Andersen *et al.* 2011), *A. niger* NRRL3 (Aguilar-Pontes *et al.* 2018), *A. niger* ATCC64974 (Laothanachareon *et al.* 2018), *A. niger* CBS513.88 (Pel *et al.* 2007), *A. niger* CBS554.65 (Ellena *et al.* 2021), *A. niger* CBS101883 (Vesth *et al.* 2018), *A. niger* ATCC13496 (Vesth *et al.* 2018), *A. niger* ATCC13157 (Vesth *et al.* 2018), and outgroup *A. welwitschiae* CBS139.54b (Vesth *et al.* 2018). The complete proteome files were used by OrthoFinder (Emms and Kelly 2019) to identify the conserved proteins that are present exactly once in each of the strains. The resulting proteins for each strain were concatenated which resulted in files containing concatenated proteins. These sequences were aligned using MAFFT (Katoh and Standley 2013). RAxML version 8 (Stamatakis 2014) was used to construct a phylogenetic tree from either 100 (phylogenetic tree Aspergillus section *Nigri*) or 1,000 (phylogenetic tree *A. niger sensu stricto*) bootstrapping replicates. The resulting tree file was visualized using iTOL version 4 (Letunic and Bork 2019).

### Plasmid construction

The primers used in this study are listed in Supplementary Table 1. The CRISPR/Cas9 plasmids were constructed as described previously (van Leeuwe *et al.* 2019). In short, CRISPR/Cas9 target sequences were chosen based on CHOPCHOP predictors (Labun *et al.* 2019) for the *fwnA* (An09g05730, NRRL3_00462), *pyrG* (An12g03570, NRRL3_03466) and *nicB* (An11g10910, NRRL3_09250) genes. Target sequences were tested with BLASTn for consistency within the 24 *A. niger sensu stricto* genomes sequenced in this study. Primers were designed to create CRISPR/Cas9 plasmids containing guide RNA targeting these genes. The resulting PCR products were digested with the restriction enzyme *Pac*I (Fermentas) and ligated into vector pFC332 (Nødvig *et al.* 2015). In addition, we used a CRISPR/Cas9 plasmid targeting the *brnA* (An14g05370, NRRL3_01040) gene that has been made previously (van Leeuwe *et al.* 2019). The complete list of plasmids used in this study can be found in Table 3.

**Table 3.** Plasmids used in this study.

Plasmid name	Description	Reference
pFC332	AMA1 sequence containing plasmid with *Aspergillus* optimized Cas9 and hygromycin selection marker	Nødvig *et al.* (2015)
pFwnA1	pFC332 plasmid containing guide RNA targeting the *fwnA* gene of *A. niger*	This study
pPyrG2	pFC332 plasmid containing guide RNA targeting the *pyrG* gene of *A. niger*	This study
pNicB1	pFC332 plasmid containing guide RNA targeting the *nicB* gene of *A. niger*	This study
pTLL40.9	pFC332 plasmid containing guide RNA targeting the *brnA* gene of *A. niger*	van Leeuwe *et al.* (2019)

### Transformation of wild-type *A. niger sensu stricto* strains

PEG-mediated *A. niger* transformations and media preparations were carried out as previously described (Arentshorst *et al.* 2012; van Leeuwe *et al.* 2019). A total of 2 µg of each CRISPR/Cas9 plasmid was used per transformation. Auxotrophic markers (*pyrG* or *nicB*) and color markers (*fwnA* or *brnA*) were introduced via CRISRP/Cas9-based genome editing. Two genetic disruptions were performed in a single transformation experiment introducing either the disruption of *brnA* and *nicB* (parent A) or the disruption of *fwnA* and *pyrG* (parent B). Protoplasts were plated on MM with sucrose (MMS) containing 200 μg/mL hygromycin and 500 µg/mL caffeine, supplemented with the required compound for the auxotrophic strains (20 µM nicotinamide for a *nicB*^*−*^ mutants and 10 mM uridine for a *pyrG*^*−*^ mutants). Transformants with a brown or fawn phenotype were selected and purified on supplemented MM plates containing 100 µg/mL hygromycin and the required supplementation. After purification on MM with supplement, the transformants were plated on MM with supplement and MM without supplement to test for the nicotinamide or uridine requirements. Transformants that had the correct conidia coloration and were unable to grow without supplement in the last purification round were harvested and used for parasexual crossings. The mutant strains generated in this study are listed in Table 2.

### Forced heterokaryon formation between *Aspergillus niger sensu stricto* strains

Heterokaryon formation was tested by mixing protoplasts of complementing parental strains and plating out the mixture of protoplasts on MM plates without supplement to select for heterokaryons. In short, protoplasts of both parents (parent A and parent B) are mixed gently and subsequently incubated in 1 mL PEG buffer for 5 min similar to the PEG-mediated transformation protocol (Arentshorst *et al.* 2012). After PEG incubation, the suspension was diluted with 2 mL STC buffer and subsequently plated on MMS plates containing 500 µg/mL caffeine, but without supplementation of nicotinamide or uridine. Since both parent A and parent B are auxotroph for different compounds, only when protoplasts of the 2 parents fuse together to form a heterokaryotic mycelium can the fungus survive.

### Diploid selection and purification

**Fig. 2.**
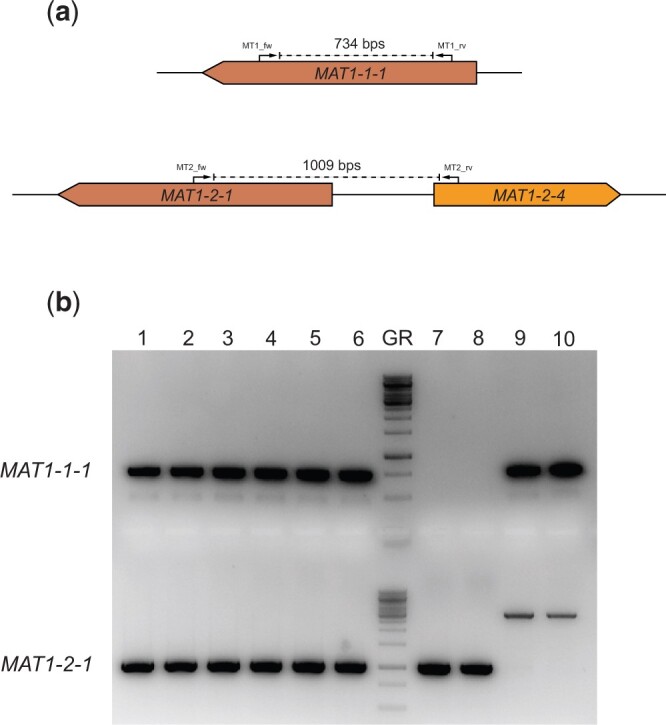
Diagnostic PCR for the presence of the mating-type genes. a) An in silico representation of the diagnostic PCRs performed to investigate presence of the mating-type genes. The primers were designed to be idiomorph-specific based on the haploid parents. If the *MAT1-1-1* gene is present, amplification with the MT1_fw and MT1_rv primers will result in a 734 bps band on the gel. If the *MAT1-2-1* gene is present, amplification with the MT2_fw and MT2_rv primers will result in a 1009 bps band on the gel. b) This gel shows the diagnostic PCR results confirming the presence of both the *MAT1-1-1* gene and the *MAT1-2-1* gene in the 6 diploid strains. Columns 1–6 are PCR products resulting from amplification on gDNA from individually obtained diploid strains SJS150.1, SJS150.2, SJS150.3, SJS151.1, SJS151.2, and SJS151.3. In the diploid strains, both PCR products are present confirming the presence of both mating-type genes. Column 7 contains the GeneRuler 1 kb DNA ladder (Thermo Scientific). Columns 8 and 9 are PCR products resulting from amplification on gDNA from *MAT1-2* containing CBS147323 parental strains (SJS111 and NS4, respectively). Columns 10 and 11 are PCR products resulting from amplification on gDNA from *MAT1-1* containing CBS147347 parental strains (SJS114 and NS8, respectively). In the haploid parental strains, only a single mating-type gene is present. Note that the CBS 147347 parental strains show off-target amplification but do not contain the *MAT1-2-1* gene.

A stable diploid strain was isolated from the heterokaryotic mycelium (Supplementary Fig. 1). A small piece of heterokaryotic mycelium was cut out from the MMS plates after 3 days of growth at 30°C and transferred to a new MM plate. This plate was incubated at 30°C for 7 days to maximize sporulation. During heterokaryotic growth, spontaneous diploid formation can take place (Shcherbakova and Rezvaia 1977). To isolate diploids, the conidia from the heterokaryon were harvested, filtered and plated in high concentrations on fresh MM plates. These conidia will only survive the fresh MM plate if genotypes of both parents are present. After 5 days of incubation at 30°C colonies with normal (non-heterokaryotic) growth and black conidia were isolated, as these are the potentially diploid strains, and plated on MEA plates. These putative diploids strains were point inoculated on MM containing 0.4 µg/mL benomyl to show the true diploidy in these strains. The mating-type loci were amplified by performing diagnostic PCR and subsequently sequenced to confirm that these stable diploids contained both mating types (Fig. 2). The conidial size was measured by taking light microscopy images and analyzing them by performing a threshold and subsequent particle analysis using Fiji (ImageJ) software (Supplementary Fig. 2). This resulted in average area for each conidial cross-section based on pixels which was converted to µm^2^ and subsequently used to calculate spore diameter assuming a perfect circle. Significance of the differences in conidial diameter was tested using a Student’s *t*-test (*P* < 0.01).

### Sclerotia formation and investigating ascospore formation

Previous observations indicated that the addition of Triton X-100 stimulated sclerotia formation in *A. niger sensu stricto* strains (Seekles, unpublished data). This ability of Triton X-100 to induce sclerotium formation in *A. niger* was assessed in laboratory strain N402. In these experiments, MEA plates with the addition of various concentrations of Triton X-100 (0%, 0.05%, 0.1%, 0.5%, and 1%) were used to find optimal concentration of Triton X-100 to induce sclerotium formation. Conidia of N402 were diluted and approximately 100 conidia were subsequently plated and distributed over the agar plate to obtain colonies derived from a single conidium. Sclerotium formation was assessed after 6 days incubation at 30°C.

Sclerotia formation of the obtained diploid strains was induced by plating conidia on MEA, potato-dextrose agar (PDA; BD Difco) and oatmeal agar (OA, BD Difco) with the addition of 1% (v/v) Triton X-100 (Sigma). In addition, sclerotia formation of both wild-type parental strains of the diploids, mixed together and plated, was assessed on MEA, OA, Czapek yeast agar (CYA), Czapek yeast agar/oatmeal agar (CYA/OA) and Wickerham’s antibiotic test medium (WATM) with the addition of 1% (v/v) Triton X-100 (Sigma). Plating was performed by point inoculation or homogenous spread of ∼100 or fewer conidia. The plates were covered in aluminum foil and left for 1–4 months at 30°C after which sclerotia formation was assessed. Sclerotia were taken from the plate and rolled over a fresh agar plate to remove conidia attached to the outside of the sclerotium. The sclerotia were cracked on top of a microscope slide, 5 µL physiological salt buffer was added, and the presence of asci/ascospores was assessed using light microscopy.

## Results

### Whole genome sequences of 24 *Aspergillus niger sensu stricto* strains

**Fig. 3.**
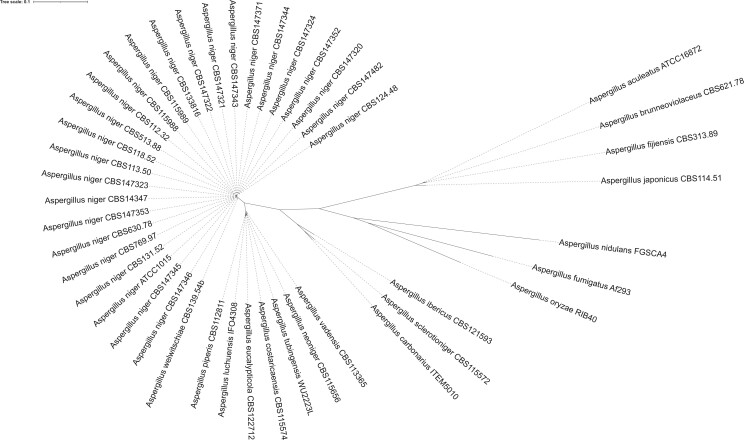
Phylogenetic tree of Aspergillus section *Nigri* strains. This phylogenetic tree was based on 3268 single-copy orthologs proteins found in all strains. Bootstrap values of all branches were calculated but not visualized to increase visibility. All branches had bootstrap values of 100, except branches between individual *A. niger sensu stricto* strains sequenced in this study (see Fig. 4) and between *A. tubingensis* and *A. costaricaensis* (bootstrap value = 69). The tree was visualized using iTOL v4 (Letunic and Bork 2019).

Twenty-four *A. niger sensu stricto* strains were studied in order to test for plasmogamy and subsequent heterokaryon compatibility. The *A. niger* strains originate from various sources from all over the world and include strains isolated from nature as well as from foods or from food-related industries (Table 1). These strains were all identified as *A. niger sensu stricto* based on partial sequencing of the calmodulin gene (Samson *et al.* 2014). The genomes of these strains were sequenced, and genes were predicted and functionally annotated. Details on the 24 genome sequences are given in Supplementary Table 2. Since the strains have been sequenced using Illumina technology, the assemblies are more fragmented than previously published assemblies. However, the gene count is comparable between the strains, and the assemblies and gene predictions are of high quality as indicated by their CEGMA and BUSCO completeness score (>98%). A phylogenetic tree was made based on 3,268 conserved proteins to verify the phylogeny of the *A. niger sensu stricto* strains when compared to other species of section *Nigri* (Fig. 3). As part of a previous study, a BLASTn search was performed in order to investigate the MAT1-1 and MAT1-2 distribution in the 24 *A. niger sensu stricto* strains and an equal distribution of mating types (12:12) was found in these strains (Ellena *et al.* 2021).

### Genome-based phylogeny of 32 *Aspergillus niger sensu stricto* strains

**Fig. 4.**
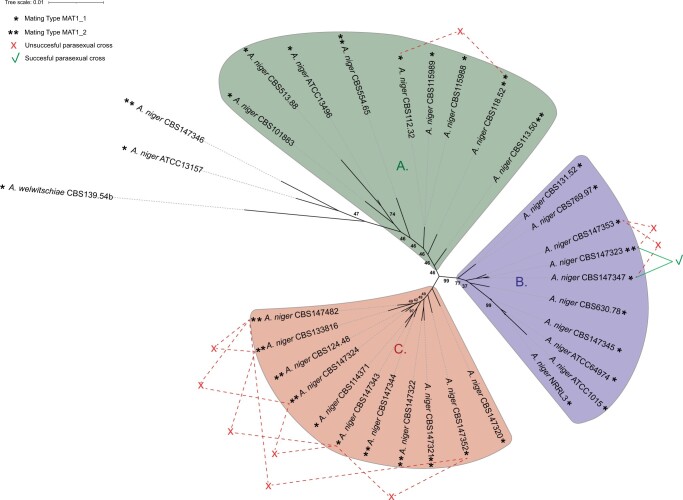
Phylogenetic tree of *A. niger sensu stricto*. This phylogenetic tree was based on 7718 single-copy orthologous proteins found in all 33 included strains. Most *A. niger sensu stricto* strains are part of 3 distinct clades, clade A, clade B, or clade C. Mating types of each strain is visualized with ∗ for the MAT1-1 locus containing genomes and ∗∗ for the MAT1-2 locus containing genomes. Heterokaryon formation via forced protoplast fusion was investigated; successful parasexual crosses are visualized by the green lines and unsuccessful parasexual crosses are visualized by the red lines. A single parasexual crossing resulted in successful heterokaryon formation, between strain CBS147323 and CBS147347 located in clade B, which are of different mating type. Bootstrap values of all branches were calculated, to improve visibility of the figure only bootstrap values indicating uncertainty (smaller than 100) are shown. The tree was visualized using iTOL v4 (Letunic and Bork 2019).

To perform successful parasexual crossings, strains need to be heterokaryon compatible. The genetic similarity between strains has a direct effect on heterokaryon compatibility. To determine the similarities between the *A. niger sensu stricto* strains, a phylogenetic tree was made based on 7,718 conserved proteins using the 24 strains sequenced in this study, as well as 8 *A. niger sensu stricto* strains obtained from literature and an *A. welwitschiae* strain as an outgroup. The tree reveals that *A. niger sensu stricto* strains can be classified in 3 distinct clades (Fig. 4). Note that some branches of the phylogenetic tree show uncertainty (bootstrap values <100), especially when determining the relative distance of certain strains within a clade to the center of the unrooted tree.

Clade A consists of 9 *A. niger sensu stricto* strains, most of which are known for their protein and enzyme production, such as *A. niger* CBS115989 (synonym NRRL3122) and its descendent *A. niger* CBS513.88 (Schäfer *et al.* 2020). Strain *A. niger* CBS115989 has been well-studied for its enzyme production (Sanzo *et al.* 2001; Manera *et al.* 2008, 2011; Abdella *et al.* 2018). In addition, the descendent strain CBS513.88, industrially used as a glucoamylase producer, has been the first whole-genome sequenced *A. niger* strain chosen based on its importance as a cell factory (Pel *et al.* 2007). Other strains in this clade are also known enzyme producers. Strain *A. niger* CBS115988 (synonym NRRL3112) has been used as an enzyme production platform in the past and present (Zaldivar-Aguero *et al.* 1997; Dartora *et al.* 2002; Abdella *et al.* 2016). *A. niger* CBS101883 (formerly known as *A. lacticoffeatus*) has not been studied extensively, however it has been used for β-glucosidase production (Cardoso *et al.* 2017). The type strain *A. niger* CBS554.65 has not been extensively studied either, although some examples of researched enzyme production exist (Gera *et al.* 2008; Chysirichote 2018). Taken together, this clade contains most if not all *A. niger sensu stricto* strains available from literature used as protein and enzyme producers by industry.

Clade B consists of 10 *A. niger sensu stricto* strains. Strains in this clade are best known for their organic acid production, such as the citric acid producer *A. niger* ATCC1015 (Andersen *et al.* 2011) and strain CBS131.51 (synonym CBS769.97, ATCC6275), which was part of a patent from 1,977 for citric acid production. Additionally, this clade contains *A. niger* NRRL3 (synonym N400) and *A. niger* ATCC64974 (synonym N402) which have been initially selected for gluconic acid production, and thereafter have extensively been used in laboratory studies (Bos *et al.* 1988; Demirci *et al.* 2021).

Clade C consists of 11 *A. niger sensu stricto* strains and none of the previously sequenced *A. niger* strains were classified in this clade. Therefore, this clade consists solely of strains sequenced in this study, many of which are isolated from food or water sources (Table 1). The genomic diversity in this clade is relatively small when compared to the differences seen between strains part of clades A and B.

The outgroup *A. welwitschiae* CBS139.54b as well as the 2 *A. niger* strains ATCC13157 and CBS147346 were considered outside of clade A. The exclusion of the 2 *A. niger* strains from clade A was based on the relatively high number of genomic variants found between these 2 *A. niger* strains and the other *A. niger* strains (see below), as well as the relatively close approximation to the outgroup *A. welwitschiae*.

### Quantification (in variants) and comparisons between *Aspergillus niger* strains

Variant calling was used to calculate the number of genomic variants present between genomes sequenced in this study. When comparing the 24 strains to publicly available strain NRRL3, an average of 6.1 variants/kb ± 2.0 (standard deviation) was found. This corresponds to an average of 213,665 variants ± 69,150 in total, when comparing all 24 strains to strain NRRL3 (Table 4). Similar results were obtained when using strain CBS147323 as a reference strain. The largest difference was found between NRRL3 and CBS147346 with genomic variant frequencies of 11.6 variants/kb, while the smallest difference was between NRRL3 and CBS147345 with only 112 genomic variants found over the whole genome.

**Table 4.** Genomic variants found between wild-type *A. niger sensu stricto* strains.

Strain name	Clade	Compared to CBS 147323 (clade B)	Compared to NRRL3 (clade B)	Compared to *Aspergillus welwitschiae* CBS 139.54.b (outgroup)
CBS 147323	B	0	149612	
CBS 147347	B	40023	148179	
CBS 147353	B	83739	149538	
CBS 630.78	B	125385	157562	
CBS 147345	B	148229	114	
CBS 131.52	B	150536	181329	
CBS 769.97	B	151239	182108	
CBS 147352	C	233388	222760	
CBS 118.52	A	238414	245379	
CBS 124.48	C	239208	231794	
CBS 147321	C	239571	231095	
CBS 113.50	A	239627	232065	
CBS 112.32	A	239627	242061	
CBS 155988	A	240185	242510	
CBS 115989	A	241224	244163	
CBS 147482	C	242026	221875	
CBS 147322	C	242026	236220	
CBS 147343	C	244034	235889	
CBS 133816	C	244298	214050	
CBS 147324	C	244303	226295	
CBS 147344	C	244594	235091	
CBS 147371	C	247056	233945	
CBS 147320	C	248958	255113	
CBS 147346	–	408752	409208	615314
**Median**		239627	231445	
**Average**		216367	213665	
**Stdev**		73157	69150	

### Heterokaryon formation between *Aspergillus niger sensu stricto* strains

Based on the phylogenetic distances between the *A. niger sensu stricto* strains, several strains were selected to perform parasexual crosses. Parasexual crosses were attempted between strains from different clades (Supplementary Table 3); however, only the attempted parasexual crosses between strains within a single clade were visualized (Fig. 4). To force heterokaryon formation and subsequent diploid formation, selected strains were genetically altered to have an auxotrophy [nicotinamide (*nicB*^*−*^) or uracil (*pyrG*^*−*^) requirement] and have conidia of altered coloration [fawn-colored (*fwnA*^*−*^) or brown-colored (*brnA*^*−*^) conidia]. The genetic alterations were made by PEG-mediated protoplast transformations using CRISPR/Cas9 technology (van Leeuwe *et al.* 2019). Since these wild-type strains contained an intact *kusA* gene, we did not include repair DNA in the transformation process, but instead selected for phenotype changes due to indels generated to escape from CRISPR/Cas9 endonuclease activity. A total of 15 strains were made, being either parent A (*brnA*^*−*^, *nicB*^*−*^) or parent B (*fwnA*^*−*^, *pyrG*^*−*^). Six strains were genetically modified to be parent A and 9 strains were modified to be parent B (Table 2). Notably, genetic alterations of strains CBS112.48 and CBS769.97 were also attempted and subsequently discontinued due to difficulties in protoplasting these strains. Heterokaryon formation was subsequently investigated using PEG-mediated protoplast fusion. Twenty-three parasexual crosses were attempted between eleven different strains. In addition, we performed 3 self-crosses between the same strain being both parent A and parent B. In short, all 3 attempted self-crosses between complementary marker strains were successful, where all 23 attempted crosses between different strains, except one, were unsuccessful (Supplementary Table 3). The single successful parasexual cross was between protoplasts of color and auxotrophic mutants of *A. niger* CBS147323 and *A. niger* CBS147347, which are located in clade B of the phylogenetic tree (Fig. 4).

### Possible heterokaryon incompatibility genes of *Aspergillus niger*

Interestingly, strains CBS147323 and CBS147347 were compatible and thus able to form heterokaryotic mycelium, but the closely related strain CBS147353 was incompatible with CBS147323 (Fig. 4). Therefore, a genetic difference between CBS147347 (compatible with CBS147323) and CBS147353 (incompatible with CBS147323) likely causes the difference in observed heterokaryon compatibility. Strain CBS147353 has ∼84,000 genomic variants compared to CBS147323 (Table 4). From these ∼84,000 variants, ∼44,000 are shared with CBS147347 when compared to CBS147323 and, therefore, are left out of the analysis as these variants could not possibly explain the difference in heterokaryon (in)compatibility observed between these strains. Still ∼40,000 variants are present in CBS147353 and absent in CBS147347 when compared to CBS147323, of which ∼9,000 variants are inside exons of genes. Therefore, the comparison between these 3 strains was limited to differences in proteins containing a HET domain, to perform an initial investigation into proteins potentially involved in heterokaryon incompatibility. The 34 HET domain containing proteins of *A. niger* NRRL3 (Aguilar-Pontes *et al.* 2018) were used to perform BLASTp analyses to find the homologs in the 3 strains CBS147323, CBS147347, and CBS14753 (Table 5). In total, 10 out of the 34 HET domain containing proteins were identical between CBS147323 and CBS147347, but different from CBS147353 and therefore could explain the observed heterokaryon compatibility difference. One of these 10 proteins is the Het-C homolog, which showed an alteration in the number of glutamine residues as has been reported before (van Diepeningen *et al.* 2009). In addition, 7 out of the 34 HET domain containing proteins were considered no actual *het* proteins active between these 3 strains, since differences in these proteins did not result in incompatibility between strains CBS147323 and CBS147347.

**Table 5.** Differences in amino acids (AAs) in HET domain containing proteins between 3 *A. niger* strains when compared to NRRL3.

Protein number	CBS 147323	CBS 147347	CBS 147353	Candidate heterokaryon incompatiblity protein between CBS 147323 and CBS 147353	Does not cause heterokaryon incompatibility
NRRL3_00449	0	0	0		
NRRL3_01616	1	1	17	NRRL3_01616	
NRRL3_01785	0	0	2	NRRL3_01785	
NRRL3_01816	9*^a^*	9*^a^*	9*^a^*		
NRRL3_02842	0	0	0		
NRRL3_02917	1	*^b^*	*^b^*		NRRL3_02917
NRRL3_03302	3	2*^a^*	2*^a^*		
NRRL3_03291	0	0	0		
NRRL3_03392	*^c^*	0	*^c^*		NRRL3_03392
NRRL3_03956	4*^a^*	4*^a^*	2	NRRL3_03956	
NRRL3_03963	0	0	0		
NRRL3_03992	*^d^*	1	*^b^*		NRRL3_03992
NRRL3_04061 (*het-c* ortholog)	0	0	−1 G	NRRL3_04061	
NRRL3_04562	0	0	*^c^*	NRRL3_04562	
NRRL3_04624	0	0	STOP	NRRL3_04624	
NRRL3_05224	0	0	6	NRRL3_05224	
NRRL3_05752	0	0	0		
NRRL3_06154	0	0	0		
NRRL3_06349	0	0	0		
NRRL3_07052	6*^a^*	6*^a^*	6*^a^*		
NRRL3_07166	*^c^*	1	*^c^*		NRRL3_07166
NRRL3_07868	0	0	0		
NRRL3_08552	FRAME_SHIFT	FRAME_SHIFT	FRAME_SHIFT		
NRRL3_08556	*^c^*	*^c^*	*^c^*		
NRRL3_08963	8*^a^*	8*^a^*	2	NRRL3_08963	
NRRL3_08976	9*^a^*	9*^a^*	2	NRRL3_08976	
NRRL3_09099	*^c^*	5	0		NRRL3_09099
NRRL3_09410	4*^a^*	4*^a^*	4*^a^*		
NRRL3_09458	16*^a^*	*^c^*	16*^a^*		NRRL3_09458
NRRL3_10072	0	0	0		
NRRL3_10361	1*^a^*	1*^a^*	1*^a^*		
NRRL3_10454	*^b^*	0	*^b^*		NRRL3_10454
NRRL3_11116	0	0	1	NRRL3_11116	
NRRL3_11636	0	0	0		

a The specific amino acid differences were equal between these strains when compared to NRRL3.

b A large gap inside this gene.

c Gene is absent.

d A big insertion inside this gene.

### Purification of a stable heterozygous diploid *Aspergillus niger* strain containing 2 mating types

The crossing of *A. niger* CBS147323 and *A. niger* CBS147347 was performed in 2 ways: CBS147323 (*brnA*^*−*^, *nicB*^*−*^) × CBS147347 (*fwnA*^*−*^, *pyrG*^*−*^) resulting in 3 independently obtained diploid strains SJS150.1, SJS150.2, and SJS150.3 and CBS147323 (*fwnA*^*−*^, *pyrG*^*−*^) × CBS147347 (*brnA*^*−*^, *nicB*^*−*^) resulting in 3 independently obtained diploid strains SJS151.1, SJS151.2, and SJS151.3 (Table 2). Three lines of evidence support that the SJS150.1-3 and SJS151.1-3 strains are true diploids.

First, the 6 independently obtained diploid strains were checked for sector formation in the presence of benomyl, since growth in the presence of benomyl forces haploidization in diploids of *Aspergillus* species (Hastie 1970). Indeed, haploidization was observed in the presence of benomyl, as shown by the sectors that displayed the original color markers again (Supplementary Fig. 1). Second, since the heterozygous diploid obtained was made between strains with different mating types, the presence of both mating-type loci was analyzed in the 6 diploid strains SJS150.1-3 and SJS151.1-3. A diagnostic PCR was performed on the genomic DNA of the diploid strains and confirmed the presence of both the *MAT1-1-1* gene and the *MAT1-2-1* gene in all 6 independently obtained diploid strains (Fig. 2). Third, conidia of diploid aspergilli are known to have an increased size (Pontecorvo *et al.* 1953). Therefore, we assessed average conidial sizes by calculating spore diameter using light microscopy comparing the diploid strain SJS150.1 with both parental strains. On average, conidia from CBS147323 and CBS147347 had diameters of 5.8 ± 0.6 and 5.5 ± 0.4 µm, respectively. In contrast, conidia from diploid SJS150.1 had an average diameter of 7.5 ± 0.6 µm. Indeed, conidia obtained from the diploid strain were significantly larger than conidia from either parent, confirmed statistically with a Student’s *t*-test (*P* < 0.01).

### Sclerotia formation on medium supplemented with Triton X-100

**Fig. 5.**
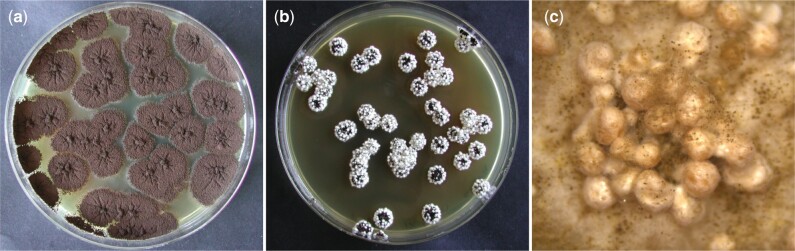
Sclerotia induction of *A. niger* N402 on MEA + Triton X-100 plates. Plates had an inner diameter of 88 mm. Here, the effect of Triton X-100 on sclerotium formation in *A. niger* N402 was assessed. Plates were inoculated by confluently plating ∼25 conidia per plate. Pictures of plates were taken after 6 days of incubation at 30°C. a) Control plate *A. niger* colonies growing on MEA for 6 days at 30°C. b) Plate containing MEA + 1% Triton X-100 (v/v). Sclerotia formation is induced in *A. niger* N402 by the addition of Triton X-100. c) Picture of a single colony from the plate shown at (b), taken after 14 days using a stereo microscope (Leica EZ4 D). The sclerotium formation in *A. niger* N402 colonies growing on MEA + 1% Triton X-100 is hyper-induced, as all individual colonies formed sclerotia on all sides of the colony.

Previous observations indicated that the addition of Triton X-100 stimulated sclerotia formation in *A. niger sensu stricto* strains (Seekles, unpublished data). The efficiency of Triton X-100 to induce sclerotium formation in *A. niger* was further assessed in laboratory strain N402. In these experiments, MEA plates with the addition of various concentrations of Triton X-100 (0%, 0.05%, 0.1%, 0.5%, and 1%) were tested (Supplementary Fig. 3). All concentrations of Triton X-100 tested were able to induce sclerotium formation; however, growing *A. niger* N402 on MEA plates containing 1% Triton X-100 (v/v) was the most effective inducer of the concentrations tested and individual colonies formed sclerotia on all sides of the colony (Fig. 5). The sclerotium induction by growth on MEA + 1% Triton X-100 plates was also assessed for various strains sequenced in this study (Supplementary Fig. 4). We noted that sclerotium formation was observed in most strains, but the degree of induction varies between strains and experiments. In addition, we tested sclerotia induction by the addition of Triton X-100 to other media, namely: MM, CM, CYA, CYA + OA, OA, WATM, and PDA. In short, sclerotia induction was observed on all these media with the addition of 1% Triton X-100, with the exception of the 2 defined media MM and CM.

**Fig. 6.**
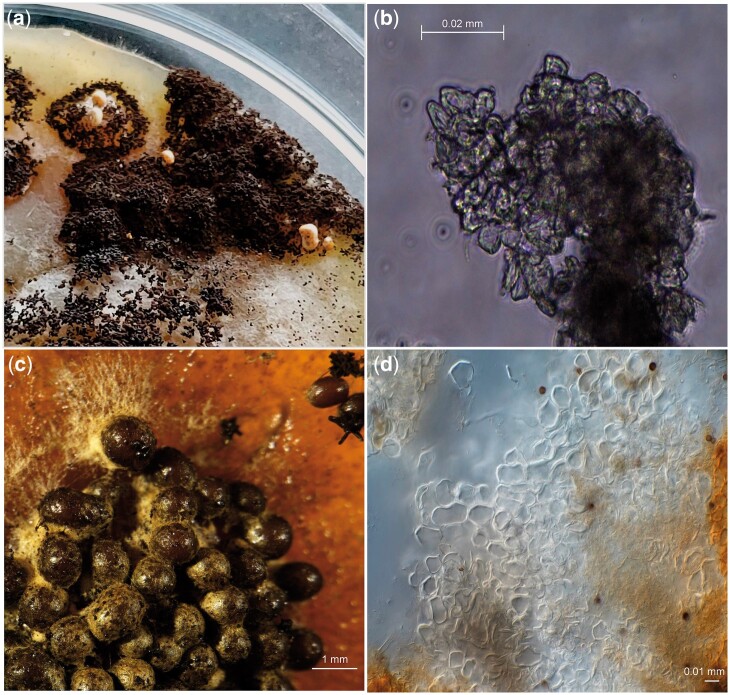
Sclerotia induction in diploid *A. niger* strains and its parental strains. Pictures were taken after 4 months of growth at 30°C in the dark in all cases. a) Diploid strains SJS150.1 and SJS151.1 were grown on sclerotia inducing media containing 1% Triton X-100. Only a single PDA and a single OA plate containing Triton X-100 showed sclerotia formation and this was limited to a small sector on the plate in both cases. b) Light microscopy was used to assess the cell structures of the obtained sclerotia obtained from the diploid strains. We found sclerotia composed of pseudoparenchymatous cells and these cells appeared to be empty, with no asci or ascospores present. c) Conidia of wild-type strains CBS147323 and CBS147347 were mixed and plated sclerotia inducing media containing 1% Triton X-100. Sclerotia formation visualized with a stereo microscope. Sclerotia formation in these 2 wild-type strains is hyper-induced as seen in N402 (Fig. 5). d) Again, light microscopy was used to assess cell structures of the obtained sclerotia from the mixed parental strains and only pseudoparenchymatous cells (of the sclerotia) without asci or ascospores were observed.

Sclerotia formation was induced in the diploid strains SJS150.1 and SJS151.1 on MEA, PDA, and OA containing 1% Triton X-100 (Fig. 6, a and b). Only a limited amount of sclerotia were obtained from the diploid strains. The sclerotia obtained were studied using light microscopy; but no (empty) asci or ascospores were observed. We noted that on the backside of the plates the regions that showed sclerotium formation also produced a brown pigment released into the media, sclerotia of *A. niger* secreting liquid of brown pigmentation has been noted before (Frisvad *et al.* 2014).

Apart from inducing sclerotia formation of the diploid strain, sclerotia were also induced in parental strains CBS147323 and CBS147347 by mixing conidia and subsequently plating them on plates containing 1% Triton X-100 in an attempt to induce the sexual reproduction between these wild-type strains (Fig. 6, c and d). We observed hyper-induction of sclerotia when conidia of parental strains CBS147323 and CBS147347 were mixed and plated together on plates containing 1% Triton X-100. Notably, the amount of sclerotia obtained after 4 months on the plates containing the mixed parental strains was considerably larger than for the plates containing the diploid strains. Unfortunately, when analyzing the sclerotia under the light microscope, no indicators of sexual reproduction (asci, ascospores) were found.

## Discussion

The sequences obtained from 24 *A. niger sensu stricto* strains are a rich resource for future research on the strain diversity within the industrially relevant species *Aspergillus niger*. All 24 wild-type strains analyzed in this study are haploid strains, which suggests that *A. niger* does not proliferate as a diploid. Further full genome comparisons between the 24 strains, focusing for example on the presence of unique genes or translocations could be a valuable future research line.

A 1:1 distribution of MAT1-1:MAT1-2 mating-type loci was previously identified in these 24 *A. niger* strains (Ellena *et al.* 2021). This finding is in agreement with an earlier report (Varga *et al.* 2014), but in contrast with various other reports that stated a skewed natural distribution in favor of the MAT1-1 locus (Pál *et al.* 2008; Mageswari *et al.* 2016). The equal distribution of mating types in wild-type strains analyzed by Ellena and colleagues suggests, or otherwise increases the likelihood of, ongoing sexual reproduction. In this study, we show that both mating types are found throughout the clades present in a phylogenetic tree (Fig. 4), again emphasizing the likely ongoing (sexual) exchange of genetic material between strains of different mating types. If sexual reproduction had been abolished in this species at a certain point during speciation, one would assume that within the branches observed in the phylogenetic tree, closely related strains would contain the same mating-type locus. The widespread heterokaryon incompatibility observed in this study and this species’ supposed asexuality raises the question how exchange of genetic material can even occur within *A. niger*, if at all, a question also raised in a previous study (Pál *et al.* 2007). Since heterokaryon incompatibility is widespread in *A. niger sensu stricto*, and sexual reproduction is known to be still possible between heterokaryon incompatible strains (Saupe 2000), it could suggest that the exchange of genetic material in *A. niger sensu stricto* occurs through sexual reproduction.

Previously, comparisons have been made between *A. niger* ATCC1015, which was selected as an optimal organic acid producing strain, and *A. niger* CBS513.88, which is used for enzyme production (Andersen *et al.* 2011; Schäfer *et al.* 2020). Therefore, it is interesting to note that *A. niger* ATCC1015 and its closely related strains are part of clade A in the phylogenetic tree whereas *A. niger* CBS513.88 and its closely related strains are part of clade B (Fig. 4). This suggests a (relatively large) genetic difference between the strains used for organic acid production and the strains used for protein production as they cluster distantly within this phylogenetic tree of *A. niger sensu stricto* strains. Previous research showed that there were potentially 3 clades within *A. niger sensu stricto* (Andersen *et al.* 2011) and we could indeed confirm these 3 clades in this study. This previous study found an unexpectedly high number of variants (on average 8 ± 16 variants/kb) between CBS513.88 and ATCC1015*.* Based on these findings, Andersen and colleagues suggest that genomes within species *A. niger sensu stricto* contain a relatively large amount of variation when compared to genomes of other filamentous fungi such as *Fusarium graminearum*. Our current findings suggest that the strains CBS513.88 and ATCC1015 are on opposite sides of the phylogenetic tree within species *A. niger sensu stricto* (Fig. 4), at least based on the 33 genomes analyzed in this study. Therefore, their genetic differences are among the highest observed within the species, and therefore relatively large compared to the average of 6.1 ± 2.0 variants/kb found between *A. niger sensu stricto* strains. These new insights suggest that *A. niger sensu stricto* stains, selected based on partial calmodulin gene sequencing results, do not have an abnormally high genetic diversity when compared to other filamentous ascomycete fungi.

The genomes of aspergilli contain the genetic information for a large number of HET domain containing proteins (30+), some orthologs to known *het* proteins from other species, that all could potentially, but not exclusively, be involved in heterokaryon incompatibility (Pál *et al.* 2007; Mori *et al.* 2019). A previous study reported widespread heterokaryon incompatibility between strains from Aspergillus section *Nigri* (van Diepeningen *et al.* 1997). Here, we report that heterokaryon incompatibility is even widespread between strains belonging to the single species *A. niger sensu stricto*. The heterokaryon compatibility between strains CBS147323 and CBS147347 provided a unique opportunity to compare the genetic make-up of these 2 strains with closely related heterokaryon incompatible strain CBS147353. Ten possible candidate HET domain containing proteins could explain the difference observed in heterokaryon incompatibility between these strains. One of these 10 candidate proteins is the Het-C ortholog that has been analyzed in *A. niger* before (van Diepeningen *et al.* 2009). We found variations in the amount of glutamine residues in the Het-C protein between the 24 *A. niger sensu stricto* strains. However, these slight variations in glutamine residues are unlikely to cause heterokaryon incompatibility as has been thoroughly described by van Diepingen and colleagues. None of the 10 candidate proteins could explain all the heterokaryon incompatibility observed between the 23 parasexual crossings attempted in this study (Supplementary Table 3). It is likely that many of the proteins containing a HET domain analyzed here do not play a role in heterokaryon incompatibility. A recent study in *A. oryzae* showed that only 4 out of 44 studied HET domain containing proteins in this species have a potential role in heterokaryon incompatibility (Mori *et al.* 2019). Indeed, heterokaryon incompatibility proteins are not limited to those containing a HET domain, as shown in *Cryphonectria parasitica* (Choi *et al.* 2012). Recent studies done in *N. crassa* show the existence of multiple heterokaryon incompatibility systems that do not require proteins containing a HET domain (Heller *et al.* 2018; Gonçalves *et al.* 2019; Daskalov *et al.* 2019). Therefore, it is probable that multiple heterokaryon incompatibility systems are active within species *A. niger sensu stricto*, only some of which involve HET domain containing proteins.

The heterozygous diploid strain described here is the first stable diploid reported between distinct haploid strains containing both mating-type systems in the heterothallic fungus *A. niger sensu stricto*. Many heterothallic ascomycetes, especially heterothallic aspergilli, show heterokaryon incompatibility between strains of different mating types, such is the case for *N. crassa*, *A. flavus* and *Aspergillus heterothallicus* (Kwon and Raper 1967; Staben 1996; Olarte *et al.* 2012). Perhaps the vegetative compatibility between strains of different mating types found in *A. niger sensu stricto* suggests the absence of an active *tol* gene mediated incompatibility system in *A. niger*, which has been described as the mediator of mating-type-associated heterokaryon incompatibility in *N. crassa* (Jacobson 1992; Shiu and Glass 1999). Indeed, the highest homology we found was with HET domain containing protein NRRL3_03963 (44.8% identity, 39.2% coverage) and the homologous region only covers the HET domain itself, indicating that there might not be a true ortholog of TOL present in *A. niger*. The availability of the diploid strain SJS150.1 opens up new possibilities to study mating-type-driven interactions for example at the levels of gene expression to analyze whether genes related to sexual reproduction are activated. Note that viability of ascospores formed from a diploid *A. niger* strain might be low, as is seen for homothallic Aspergillus species such as *A. nidulans* (Pontecorvo *et al.* 1953; Elliott 1960; Garber *et al.* 1961). The *A. niger* diploid strain enables the possibility to test for a broad range of environments that might trigger sexual reproduction.

## Data availability

Strains and plasmids used are available upon request. Supplementary Fig. 1 contains a detailed overview of the purification of the diploid strains. Supplementary Fig. 2 shows the size differences between haploid and diploid conidia of *A. niger*. Supplementary Fig. 3 shows the effectiveness of Triton X-100 as inducing agent for the formation of sclerotia in N402. Supplementary Fig. 4 shows the effectiveness of Triton X-100 as inducing agent for the formation of sclerotia in various wild-type *A. niger sensu stricto* strains. Supplementary Table 1 lists primers used in this study. Supplementary Table 2 gives an overview of the overall quality of the genomes sequenced in this study. Supplementary Table 3 lists the total parasexual crosses attempted in this study. The genome assemblies and predicted genes that were sequenced in this study are available in NCBI GenBank under BioProject ID PRJNA743902. The annotated genomes can be analyzed interactively on https://fungalgenomics.science.uu.nl/.

Supplemental material is available at *G3* online.

## Supplementary Material

jkac124_Supplementary_Material

jkac124_Table_S1

jkac124_Table_S2

jkac124_Table_S3

jkac124_Figure_S1

jkac124_Figure_S2

jkac124_Figure_S3

jkac124_Figure_S4
