# Supplementary material for: Genome sequences of 24 Aspergillus niger sensu stricto strains to study strain diversity, heterokaryon compatibility, and sexual reproduction
Source: G3 (Bethesda). 2022 May 24;12(7):jkac124. doi: 10.1093/g3journal/jkac124 (PMC9258588; doi:10.1093/g3journal/jkac124)
Supplement: jkac124_Supplementary_Material [file jkac124_supplementary_material.docx]

**Figure S1. The isolation of a heterozygous diploid of *A. niger* containing both mating-types. A.** Heterokaryons grown on MMS without supplementation for 3 days at 30°C. Growth only occurs after successful protoplast fusion and heterokaryon formation. A slice of agar containing a single heterokaryon was transported to a new MM plate without supplement **B.** Heterokaryon grown on MM without supplementation for 7 days at 30°C. Conidia were harvested, filtered and subsequently plated on new MM plates without supplementation. **C.** Colonies formed after plating conidia obtained from heterokaryon. Diploid strain can be recognized by fast(er) growth and black conidia as shown by the red arrow. **D.** The Diploid colony was plated on MEA and grown for 5 days. Conidia were harvested. **E.** Diploid conidia were point inoculated on MM + 0.4 µg/ml benomyl. Haploidization is seen where sectors contain the original fawn colored conidia (yellow arrow) or brown colored conidia (brown arrow).

**Figure S2. Determination of size difference between diploid and haploid conidia of *A. niger sensu stricto*.** Light microscopy images were taken while using a 400x magnification in all cases as seen in the left column. Images were processed using FIJI (ImageJ) software by manually adjusting thresholds until loose conidia were colored red. A mask was created of isolated conidia by manually adjusting the size and circularity of the particles to be analyzed. The black areas in the masked images on the right column represent the area of conidia measured. Sizes of the particles were measured based on pixel amounts and subsequently converted diameter in µm assuming a perfect circle. On average, conidia from CBS147323 and CBS147347 had diameters of 5.8 ± 0.6 µm and 5.5 ± 0.4 µm, respectively. In contrast, conidia from diploid SJS150.1 had a diameter of 7.5 ± 0.6 µm.

**Figure S3. Sclerotia induction of *A. niger* N402 by different concentrations of Triton X-100.** Approximately 25 conidia of *A. niger* were plated on MEA + Triton X-100 plates, where Triton X-100 concentrations were varied. Plates were grown for 6 days at 28°C. **A.** MEA + 0% Triton X-100 **B.** MEA + 0.05% Triton X-100 **C.** MEA + 0.1% Triton X-100 **D.** MEA + 0.5% Triton X-100 **E.** MEA + 1% Triton X-100**.**

**Figure S4. The induction of sclerotia in *A. niger sensu stricto* strains on MEA + 1% Triton X-100.** Approximately 25 conidia were plated per plate. Plates were incubated at 30°C for 5 days in a closed incubator (not opened throughout the experiment) for both conditions. Sclerotium formation was always more successful when Triton X-100 was added. Sclerotium formation was observed in the strains N402, N400 (synonym NRRL3, the parental strain of N402), CBS115989, CBS147323, CBS147345 and CBS147346.
