# Supplementary figures and images for: Genome sequences of 24 Aspergillus niger sensu stricto strains to study strain diversity, heterokaryon compatibility, and sexual reproduction
Source: G3 (Bethesda). 2022 May 24;12(7):jkac124. doi: 10.1093/g3journal/jkac124 (PMC9258588; doi:10.1093/g3journal/jkac124)

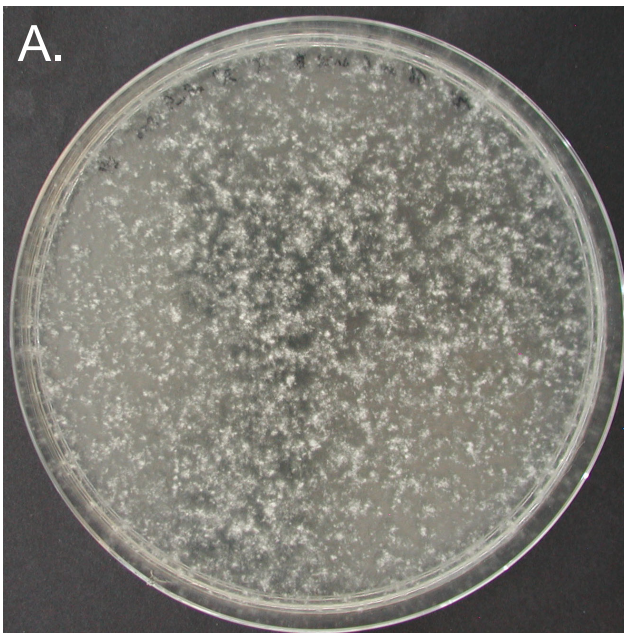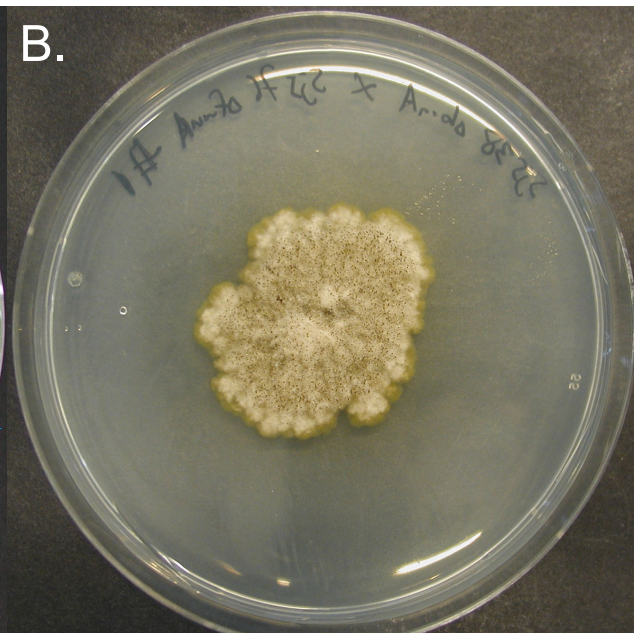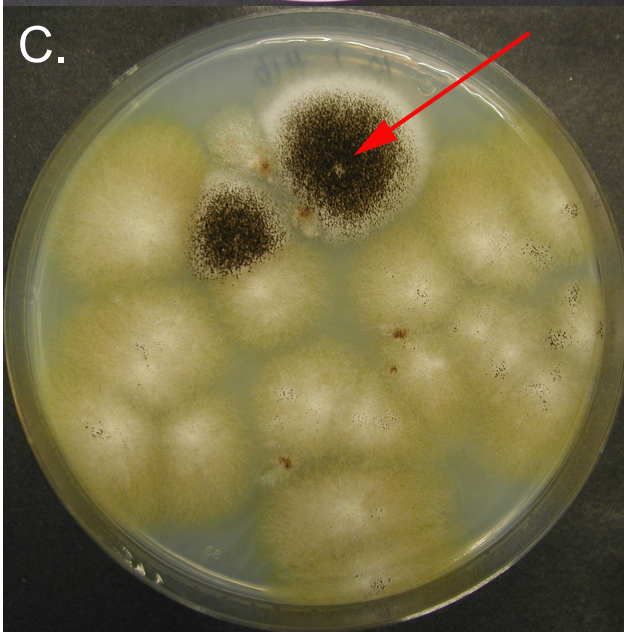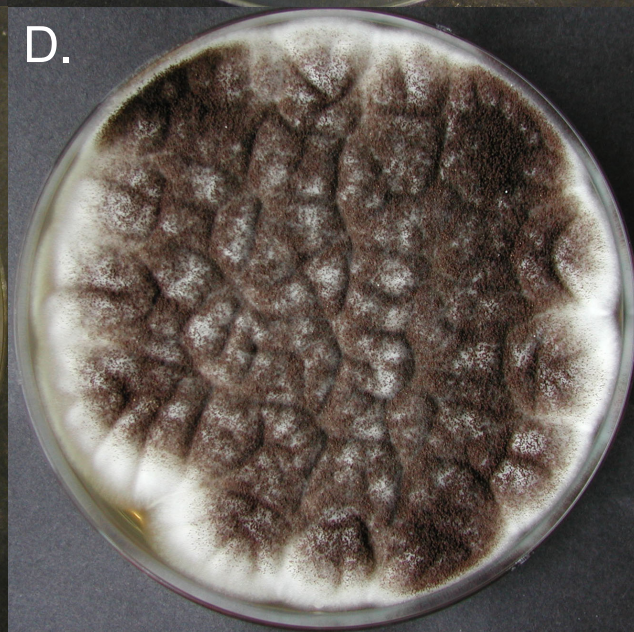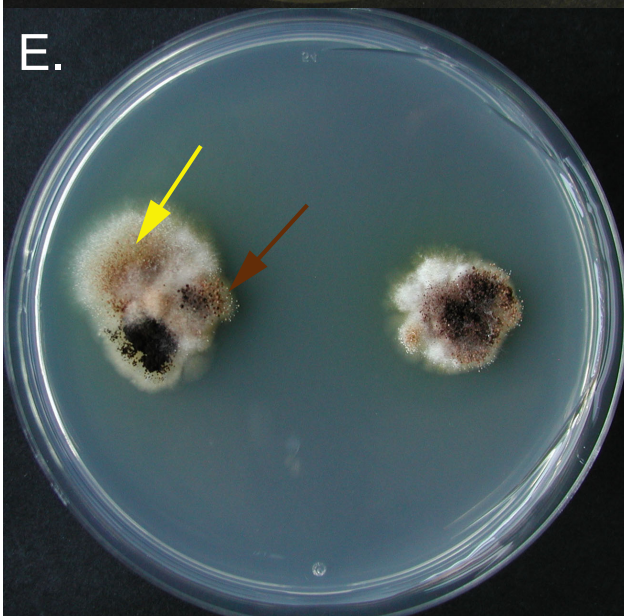

Supplement: jkac124_Figure_S1 [file jkac124_figure_s1.pdf]

Light microscopy (400x)

Masked images

CBS 147323

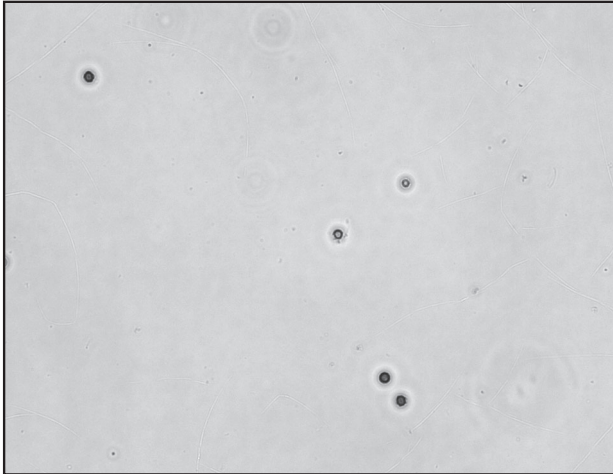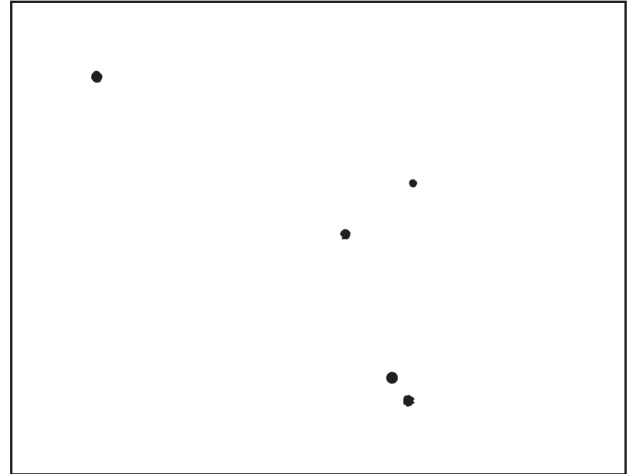

CBS 147347

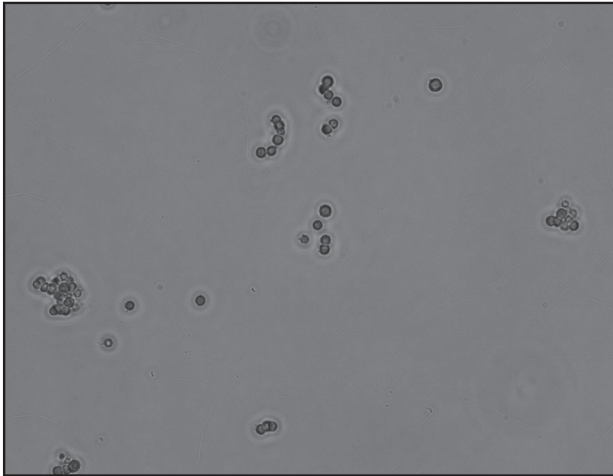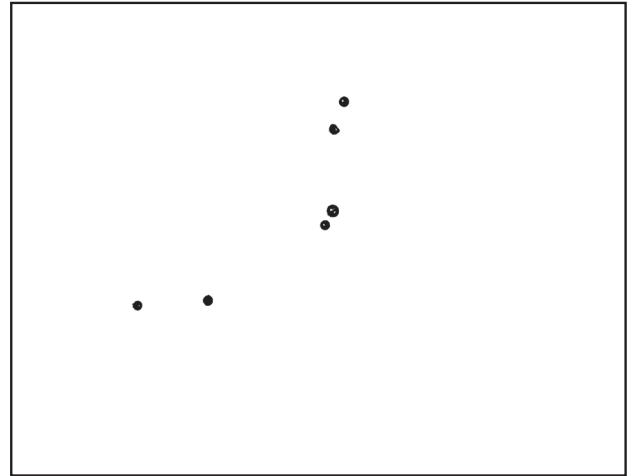

SJS150.1

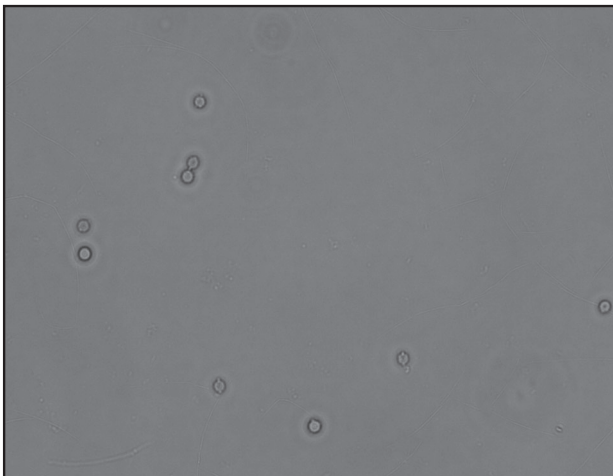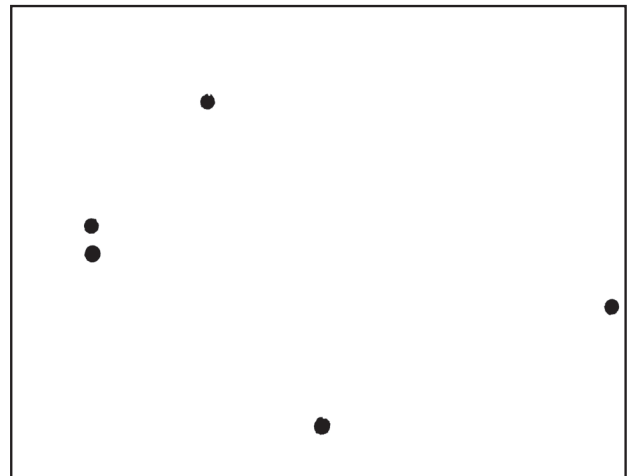

Supplement: jkac124_Figure_S2 [file jkac124_figure_s2.pdf]

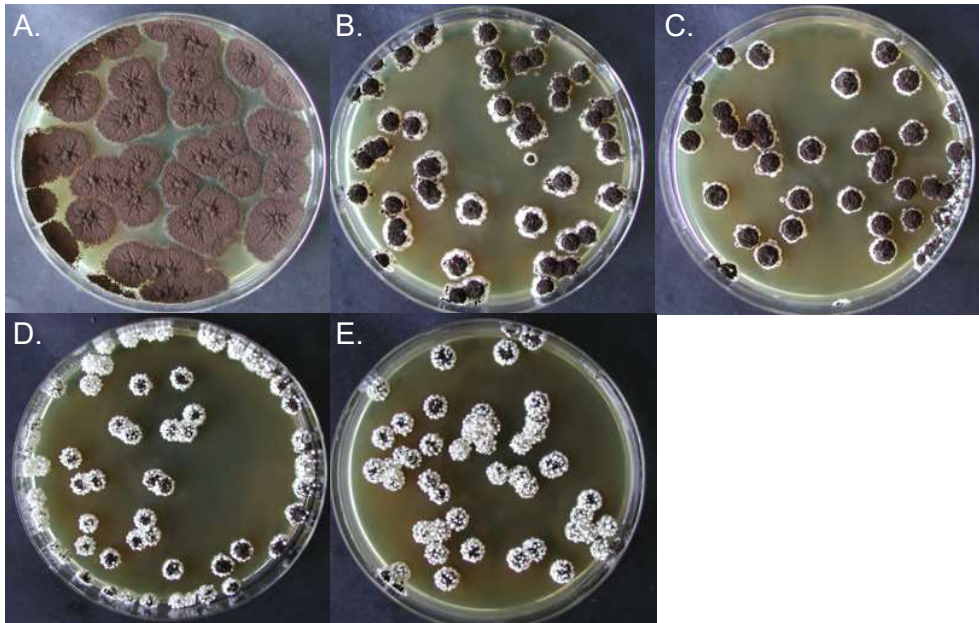

Supplement: jkac124_Figure_S3 [file jkac124_figure_s3.pdf]

Control

+1% triton

N402

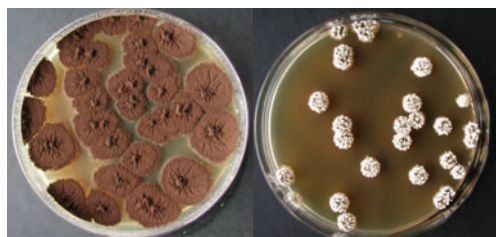

N400

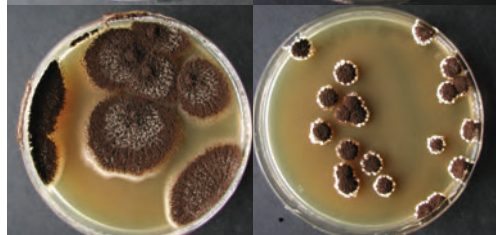

CBS 124.48

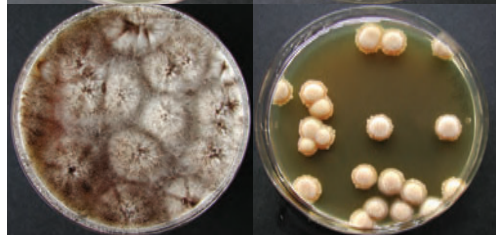

CBS 112.32

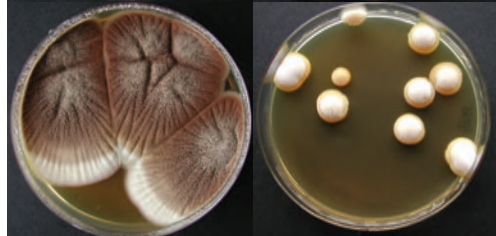

CBS 769.97

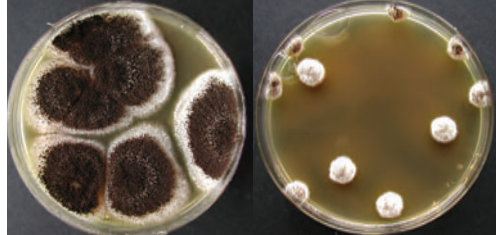

Control

+1% triton

CBS 147323

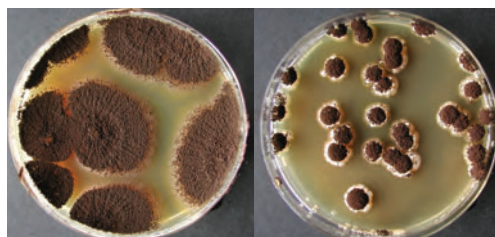

CBS 115989

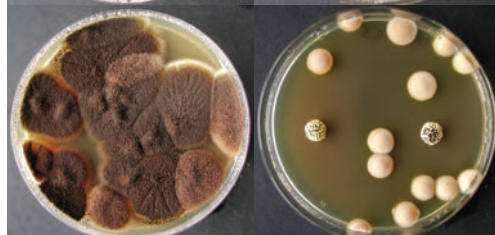

CBS 147346

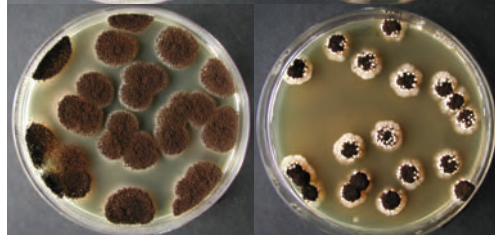

CBS 147347

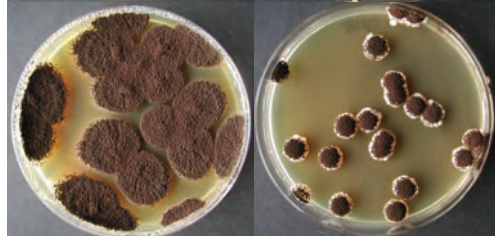

CBS 147345

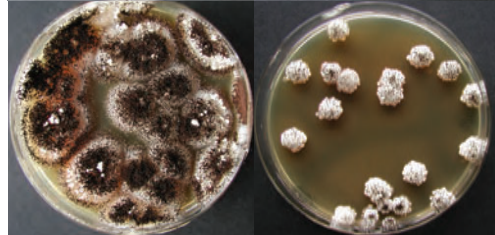

Supplement: jkac124_Figure_S4 [file jkac124_figure_s4.pdf]
